# Psychopathological and Psychosocial Risk Profile, Styles of Interaction and Mentalization of Adolescent and Young Mother–Infant Dyads

**DOI:** 10.3390/ijerph19084737

**Published:** 2022-04-14

**Authors:** Elena Ierardi, Alessandro Albizzati, Margherita Moioli, Cristina Riva Crugnola

**Affiliations:** 1 Department of Psychology, University of Milano-Bicocca, Piazza dell’Ateneo Nuovo 1, 20126 Milan, Italy; cristina.riva-crugnola@unimib.it; 2 Neuropsychiatric Unit 2, ASST Santi Paolo e Carlo, 20142 Milan, Italy; alessandro.albizzati@asst-santipaolocarlo.it (A.A.); margherita.moioli@asst-santipaolocarlo.it (M.M.)

**Keywords:** adolescent mothers, mother–infant interaction, risk factors, adverse childhood experiences, psychopathological problems

## Abstract

This study examined the psychopathological and psychosocial risk profile and the quality of mother–infant interaction in 98 adolescent and young mother–infant dyads. At their infant’s age of 3 months, mothers filled in a socio-demographic form and completed a test battery: EPDS for depression, STAY-I for anxiety, PSI-SF for parenting stress, MPSS for social support, AAI for maternal attachment and reflective functioning, CECA for adverse childhood experiences, Care-Index and Mind-mindedness coding system for mother–infant interaction. Results showed that motherhood in adolescence was associated with several psychosocial risk factors. Adolescent and young mothers have depression (25%), anxiety (29%) and insecure attachment (65%), with low reflective functioning, of whom 18% have disorganized attachment. A total of 54% of the mothers had at least one adverse childhood experience. Furthermore, adolescent mothers had low sensitivity and mind-mindedness and high intrusiveness, and their infant had low responsiveness and high passive behaviors. Mothers under 18 have experienced more sexual abuse, are more likely to be single and have been followed by child social services more than mothers aged 18–21. Adolescent mothers have a high-risk psychopathological and psychosocial profile that affects their ability to mentalize and build an adequate relationship with the child. It appears to be important to support the adolescent mother–child relationship.

## 1. Introduction

Younger mothers can be considered a disadvantaged group and at risk of experiencing not only a range of negative short outcomes but also unfavorable long-term mental health outcomes [1,2].

Adolescent and young motherhood in Italy accounts for around 1.2% of all births each year [3], a percentage which is lower than that in other countries such as the US or the UK where it is a significant phenomenon, accounting for between 11 and 14% of total births [4]. Becoming a mother at a young age is a stressful experience, involving many more challenges than is the case with adult mothers. Young women must manage multiple significant life changes at the same time: transition to adulthood, involving individuation from parental figures [5] and transition to parenthood, which involves the nurturing of an infant who is greatly dependent on her for his or her physical and emotional needs [6,7]. During pregnancy and the post-partum period, young mothers can have mixed emotions, including joy and worry, about their new responsibility and may feel alone and isolated, which exacerbates their feelings of vulnerability and low self-esteem [8]. At the same time, cognitive and neurophysiological development in adolescent mothers still has to be completed [9]. Such immaturity may influence adolescent mothers, making them less cognitively competent with regard to taking on their parental role (cognitive readiness to parent) and to knowledge about the abilities of the child in the different stages of development [10].

The negative impact of adolescent pregnancy is not only linked to maternal age but also to the various social, psychological, individual, relational, economic, and environmental risk factors often associated with it interdependently [11]. Adolescent and young mothers often have a socio-economic disadvantage, low levels of education, school difficulties and interruption of studies, lack of social support, unstable relationships with partners and, consequently, a greater likelihood of being a single parent, of having a multiproblematic family of origin with an absent father, a history of young parenthood, above all on the part of the mother, and an unwanted or unplanned pregnancy [10,12,13,14,15,16].

Another risk factor is a higher number of adverse cumulative childhood experiences of physical, sexual and emotional abuse in adolescent mothers than in adult mothers [17,18,19]. These experiences typically occur in a dysfunctional family and social context [20]. Various studies show that adolescent girls with a history of maltreatment have a higher risk for early pregnancy [21,22,23]. This may lead to an increase on the part of adolescent mothers in maltreatment of and hostile behavior towards the infant [24], with intergenerational transmission of traumatic experiences from mother to child [25]. The types of maltreatment which adolescent mothers alleged they had experienced during childhood were related to the type of maltreatment they perpetrated [26]. A recent study [26] reported that 62% of adolescent mothers who maltreated their children had prior experience with child protective services.

In addition to traumatic experiences many risk characteristics of the family context or of the young girls can often be traced back to before pregnancy and are predictive factors thereof. Studies have identified conduct disorders, aggressive and delinquent behavior and bullying in pre-adolescence, depression prior to pregnancy, and having a mother who gave birth during adolescence [27,28,29]. The condition of disadvantage for adolescent mothers is therefore already present before they become mothers [30,31]. O’Flaherty et al. [32] also showed that it is the disadvantaged condition of the young women prior to pregnancy more than the effects of teen motherhood which gives rise to associations between young motherhood and later life mental health.

Multiproblematic family contexts and a history of adverse childhood experiences affect attachment models. There is a greater prevalence among adolescent mothers of insecure attachment models, with a higher risk of developing insecure and unresolved/disorganized attachment models, than among adult mothers [21,33]. Capacity for mentalization in young mothers is also low in the level of adult reflective functioning [34], parental reflective functioning [35] and mind-mindedness [34,36,37]. As is well-known, a good capacity for mentalization is often associated with maternal sensitivity and predicts secure child attachment [38].

All these risk factors which characterize young motherhood increase the risk for adolescent and young mothers in the perinatal period of distress and psychopathological problems. Several studies indicate that adolescent mothers have an up to 50% greater probability of developing post-partum depression; they also have a higher degree of emotional distress, post-traumatic stress disorder (PTSD), substance abuse, and of being exposed to adverse experiences and Intimate Partner Violence (IPV) than adult mothers [39,40,41]. However, they have lower levels of self-esteem and self-efficacy than adult mothers [42].

The psychological vulnerability caused by potential conflict between the various developmental tasks and the multiplicity of risk factors which are often associated with adolescent motherhood may have a strong influence on the quality of parenting and of parental responsiveness and on the mother–infant relationship from the first months on. Adolescent and young mothers are less responsive and empathic [43], adopt more hostile and intrusive or detached behaviors [44,45], and are also more likely to adopt harsh parenting, accompanied by both physical and verbal abuse [24]. They have difficulty understanding the needs of their infants and have little knowledge of their stages of development [46], using more instrumental behavior in caring for their children [47]. Adolescent mothers are also less verbally stimulating and less vocally responsive and interact with their infants in a peer-like manner [48,49], compared to adult mothers. They show poorer ability in scaffolding the activity of their infants [50]. Moreover adolescent mothers show poor emotional availability [50], less structuring of their infant’s activity [44], less adequate dyadic emotional regulation, and have more difficulty in regulating their own and their infants’ negative emotion states compared to adult mothers [51].

For these reasons the children of adolescent and young mothers are at risk for adverse developmental trajectories such as insecure and disorganized attachment [52,53]; often suffer maltreatment, abuse and neglect [54]; and have delays in their psychomotor, especially linguistic and cognitive, development [16,55,56]. They also display less ability in affective communication [57]. When the children become teenagers, they are at a high risk of becoming parents themselves in adolescence and of having low levels of education, low-income levels, mental health problems, substance abuse and delinquency problems and of exhibiting antisocial behavior [14,58,59]. At the same time, early motherhood can limit the subsequent life opportunities of young women [60], leading them being underemployed during adulthood and giving rise to a high probability that they have depressive symptoms and substance abuse problems and experience social isolation [17,61,62].

However, if young mothers are adequately supported by friendly and non-stigmatizing primary care [46,63] and by dedicated intervention programs, pregnancy at an early age may become an opportunity for change. The young mother can experience the birth of her infant as a turning point with respect to defining her identity, increasing her investment in herself and reducing possible risky behavior adopted prior to the birth, e.g., substance abuse, promiscuity, etc.

Few studies have examined adolescent and young fathers. This is, in part, because they are often not very present since in most cases the young mothers live with their families of origin and are very unlikely to form a new family unit. When such fathers are present, they do not appear very emotionally involved with the child or the adolescent mother [64]. Concerning risk factors, the characteristics of young fathers are similar to those of adolescent mothers: most come from disadvantaged socio-economic contexts, have earlier sexual relations and more infant negative experiences than adult fathers; they also have a low level of education and little possibility of finding employment [65]. Adolescent fathers tend to have great difficulties at psychological, emotional and social levels, with depressive states and aggressive delinquent behavior as well as alcohol and drug abuse, which often continue after childbirth [66].

While there are numerous studies on risk factors in young motherhood, there are, to our knowledge, no studies which have focused on the combination of socio-demographic risk factors, considering at the same time the interaction between mother and infant in the first months. There has also been no study on state and trait anxiety in adolescent mothers and only a few studies [34,35,38] have examined their capacity for mentalization.

### The Current Study

The first objective of this study is to outline the psychopathological and psychosocial risk profile of adolescent mothers and young mothers up to 21 years of age in an Italian sample, examining all risk factors in an interconnected way and bridging the gap in the literature. Firstly, we investigate the socio-demographic characteristics that can be a risk factor: psychological and psychopathological problems in the postpartum period such as maternal anxiety and depression, parenting stress, perceived social support, presence of adverse experiences in maternal history, reflective functioning and maternal attachment. Secondly, at the infant’s age of 3 months, we examine the interaction styles of mother and infant and maternal mind-mindedness. We hypothesize that adolescent mothers and young mothers have low socioeconomic levels, multi-problem family backgrounds and low levels of education. We also hypothesize, on the basis of the literature, that they have a high level of depression and anxiety, parenting stress, insecure attachment patterns, several adverse childhood experiences, low mentalization levels and low maternal and infant sensitivity. Moreover, we hypothesize that maternal depression, anxiety and parenting stress are intercorrelated and maternal depression and anxiety are associate with maternal and infant style of interaction.

The second objective is to identify any differences between mothers under 18 years and young mothers (18–21 years) with respect to the risk factors considered. We hypothesis that mothers under 18 years have more psychosocial risk factors than young mothers.

The third objective is to identify whether one or more risk factors are more predictive of low sensitivity and poor mentalization in adolescent mother–infant interaction. Since there are no studies that consider all risk factors, to our knowledge, the third objective was conducted on at an exploratory level.

## 2. Method

### 2.1. Participants

A total of 98 adolescent and young mothers recruited from the “*Accompagnamento alla genitorialità in adolescenza*” (SAGA—Accompanying Parenting in Adolescence) Service at the San Paolo Hospital of Milan when the infant reached 3 months. SAGA is a service that helps adolescent and young mothers aged up to 21 years with an attachment-based intervention program consisting of psychological support, child developmental guidance, and video feedback [7].

Inclusion criteria were: mother’s adequate knowledge of the Italian language; maternal age range between 14 and 21; uneventful delivery; infants born with no medical complications and physically healthy; and primipara mothers. Exclusion criteria were low prematurity and twin birth.

Mothers (and their babies) were contacted after the baby was born; the assessment was started when the infants were 3 months of age before the intervention program. The research was proposed to 150 mothers, of which 98 mother–infant dyads (65%) participated in the study (male children = 49); 15% refused to attend; and 20% canceled their appointments. Data for psychopathological risk and mind-mindedness were available for half of the mothers. The recruited group had similar characteristics to that of the mothers who were not recruited.

At infant age of 3 months, mothers were given an ad hoc module for the collection of socio-demographic information, questionnaires and interviews. Mother–infant interactions were videotaped for approximately 5 min (M = 5.02; SD = 0.40) in a hospital room with children’s toys and pillows, framed sideways to codify the behavior and facial expressions of both members of the dyad. Mothers were asked to interact with their children as they were used to at home. Mother–infant interactions were coded with Care-Index and Mind-Mindedness system to evaluate maternal and infant styles of interaction and maternal mentalization.

The institutional review board of the ASST Santi Paolo e Carlo of Milan approved the study protocol. All subjects gave their written informed consent.

### 2.2. Measures

#### 2.2.1. Socio-Demographic Profile

Ad hoc anamnestic form was created to evaluate socio-demographic characteristics such as: socio-economic level, level of education, with whom they live or living in a residential community, desired/unwanted pregnancy, presence/absence of the partner, divorce or separation of parents, history of parenthood at a young age, unemployment, and presence of child social services.

#### 2.2.2. Postpartum Depression

The Edinburgh Postnatal Depression Scale (EPDS) [67] is a 10-item self-report questionnaire that has been used to assess the presence of depressive symptoms during postpartum. In this study, we used the clinical cut-off (between 9 and 12 medium level, 13 or more high level) indicated by the Italian validation [67]. In our study, internal consistency for the EPDS was good (Cronbach’s α = 0.77).

#### 2.2.3. Anxiety

State Trait Anxiety Scale (STAI-Y) [68] is a 40-item self-report questionnaire that has been used to assess maternal anxiety. It is composed of two scales: State Anxiety, regarding the current state of anxiety, and Trait Anxiety, regarding the type of anxiety which is characteristic of the personality of the subject. In the current study, we used a clinical cut-off > 39 for state anxiety and a cut-off > 42 for trait anxiety. In our study, internal consistency for the State Anxiety scale (Cronbach’s α = 0.86) and for the Trait Anxiety scale (Cronbach’s α = 0.65) was good.

#### 2.2.4. Parenting Stress

Parenting Stress Index-Short Form (PSI-SF) [69] is a self-report questionnaire used for early identification of factors which may compromise normal infant development, in particular parenting stress. It is composed of 4 scales: (1) Parenting Distress: level of distress of the parent caused by personal factors linked to the parental role; (2) Dysfunctional parent-infant interaction: focused on the parent’s perception that the infant does not meet his/her expectations; (3) Difficult infant: focused on several characteristics of the child, which make them easy or difficult to handle and which stem from their temperament; and (4) Total Stress. In our study, internal consistency for the PSI total score was excellent (Cronbach’s α = 0.95).

#### 2.2.5. Social Support

Multidimensional Scale of Perceived Social Support (MSPSS) [70] is a self-assessment questionnaire which investigates the perceived level of social support from 3 sources: Family, Friends, and a Significant Other. The scale is comprised of a total of 12 items, with 4 items for each subscale M. The total scores divided by 12 items put the subject into 3 groups on the basis of their scores (trichotomize) and designated the lowest group as low perceived support, the middle group as medium perceived support, and the high group as high perceived support. In our study, MSPSS scale showed good reliability (α = 0.96).

#### 2.2.6. Maternal Attachment

Adult Attachment Interview (AAI) [71] is a semi-structured interview that has been used to examine the adult attachment models, exploring the interviewees’ relations with their parents as children. According to the Main coding system [72], based on 9-point scales, each interview was assessed for the following categories: Secure/Autonomous (F), involves consistent and objective narration of attachment experiences and their assessment; Dismissing (Ds), involves inconsistent narration of attachment experiences with idealization of attachment figures; Preoccupied (E), involves inconsistent narration characterized by vagueness and prolixity together with worry and/or anger expressed towards attachment figures; Unresolved/Disorganized (U), involves failure to process traumatic episodes (maltreatment, abuse, etc.) and mourning; and Cannot Classify (CC), involves the co-presence of contradictory mental states with regard to attachment.

The AAI coding system provides scales which are related to inferred experiences, and scales related to state of mind with regard to attachment figures. In our study we used the Coherence of Mind scale, which is assessed as the degree to which an individual is relevant, understandable, coherent and concise in their descriptions of childhood attachment memories, and how the expressed beliefs are consistent with reality. Coherence of Mind is associated with the Secure Autonomous category [73].

Concordance between the two coders, calculated on 20% of the interviews, for the four-way classifications was k = 0.72 and for the two-way classifications (secure versus insecure) was k = 1.00.

#### 2.2.7. Reflective Functioning

The Reflective Functioning scale (RFS) [74] applied to the Adult Attachment Interview has been used to assess adult’s mentalization ability to reflect on his own and another’s experiences in terms of mental states and emotions. Reflective function are scored on a scale from −1 to 9: Negative RF (−1) indicates subjects who are confused or hostile and refuse any reflection; Lacking in RF (1), indicates that reflective function is totally or almost totally absent; Questionable or Low RF (3) covers subjects who display some evidence of awareness of mental states, at rudimentary level; Ordinary RF (5) refers to subject that have consistent, though simple, capacity to reflect on attachment figures and on their own mind; Marked RF (7) indicates subject who demonstrate awareness of the nature of mental states for the entire interview; Exceptional RF (9) covers subjects who have exceptional and sophisticate ability to recognize causal relation in which mental states are used. Reliability between coders was calculated on 20% of the interviews through the intraclass correlation coefficient and was ICC = 0.77.

#### 2.2.8. Adverse Childhood Experiences

Childhood Experience of Care and Abuse (CECA) [75] has been used to evaluate adverse childhood experiences before age 17 resulting from the AAI interviews of the study participants. The main scales include are neglect, antipathy, physical and psychological abuse from different parent figures as well as sexual abuse from any perpetrator. Each type of maltreatment was rated on a 4-point severity scale (1 = marked, 2 = moderate, 3 = some, 4 = little or none), according to predetermined criteria and manualized threshold examples. These scales were also dichotomized into severe (marked/moderate) and non-severe (little or none) as in previous studies using the CECA [76]. A summary index of childhood adversity involving the peak experience of ‘marked’ or ‘moderate’ neglect, antipathy and abuse in childhood was used [76]. CECA coding system, provide, in addition to the main scales mentioned above, also other scales, such as loss of parents, parental control, level of discord between parents, violence between parents, role reversal and parent mental health. In our study, the index of childhood adversity scale showed moderate internal consistency (α = 0.65).

#### 2.2.9. Mother–Infant Styles of Interaction

Care-Index [77] has been used to codify mother–infant interaction on the basis of 7 behavioral characteristics: facial expressions, vocal expressions, body position and contact, affection, turn-taking, control and choice of activity. Parental styles of interaction are assessed on three scales: Sensitive with expression of positive effects and responsiveness towards the emotions and actions of the child; Controlling with hostility and intrusiveness towards the activities of the child; Unresponsive with physical and emotional detachment. The styles of interaction of the child are assessed on four scales: Cooperative with expression of positive emotions and acceptance of actions undertaken by the parent; Compulsive–Compliance with cautious and inhibited behavior and a compliant approach towards the parent; Difficult with resistance to proposals of the parent; Passive with physical and emotional withdrawal.

Each scale is assessed on the scores from 0 to 14. regarding maternal sensitivity, the range of scores 0–4 is considered at high risk; the range of scores 5–6 is considered marginally adequate; 7–10 indicates adequate sensitivity and 11–14 is considered very good sensitivity. Reliability between observers was calculated on 20% of the observations of the dyads through the intraclass correlation coefficient and was ICC = 0.81 for maternal behavior and ICC = 0.73 for infant behavior.

#### 2.2.10. Mind-Mindedness

Mind-Mindedness coding system [78] has been used to evaluate maternal mind-mindedness during a video-recorded 5 min free-play session. Mothers’ speech during the sessions was transcribed verbatim and was divided into: comments not related to the infant’s mind or emotion (Not Mind-Related) and comments that included an internal-state term related to the infant’s mind or emotion (Mind-Related comments). Mind-related comments included references to wishes and desires, mental states, mental processes, emotions, attempts to manipulate people’s beliefs and comments where the mother “put words into her infant’s mouth”. A mind-related comment was also classified as an appropriate mind-related comment if one or more of the following conditions were met: (a) the independent coder agreed with the mother’s reading of her infant’s internal state, (b) the internal state comment linked the infant’s current activity to similar events in the past or future, (c) the internal state comment served to clarify how to proceed if there was a lull in the interaction or (d) the mother voiced (using the first person) what the infant might say if he/she could speak.

To control maternal verbosity, the mind-mindedness score was the number of mental descriptors expressed as a proportion of the total number of descriptors used. Higher proportional scores indicated greater mind-mindedness. Reliability between observers was calculated on 20% of the transcripts trough inter-rater reliability and was K = 0.93 for mind-related comments, and K = 0.90 for appropriate mind-related comments.

## 3. Data Analysis

SPSS 27 was used for statistical analysis. We managed the missing data with listwise deletion. Descriptive statistics were calculated with respect to demographic characteristics; the Pearson correlation for the continuous variables and the Chi-square test (or Fisher’s exact tests) for nominal variables were applied to identify the relationship between the variables. *t*-test was used to analyze the difference between mothers under 18 (14–17 years old) and young mothers (18–21 years old) on all variables. Regression multiple analysis was used to evaluate the effect of risk factors on mother–infant interaction styles and maternal mind-mindedness. A power analysis indicated that a sample of 98 participants was sufficient to detect a medium effect size with a power of 0.86 (α = 0.05).

## 4. Results

### 4.1. Socio-Demographic Characteristics

Table 1 showed the socio-demographic risk profile of adolescent and young mothers. Mothers had a mean age of 18.46 (SD = 1.99) (range 14–21 years old). In most cases adolescent mothers came from a low and medium-low socio-economic background, had a low level of education and in more than half of the cases did not work or attend school. Furthermore, nearly all mothers had a history of parenting in adolescence from one parent and in one third of cases they did not have a partner. More than 50% of mothers lived with their parents. In more than 70% the pregnancy was unwanted. A total of 30% of the young mothers had been followed by child social services and had been in a residential mother and baby community.

Moreover, low socio-economic level was associated with a low level of education, (χ^2^ = 4.20; *p* = 0.040) and unwanted pregnancy (χ^2^ = 5.02; *p* = 0.035) but was not associated with adverse childhood experiences (χ^2^ = 0.13; *p* = 0.71).

### 4.2. Psychopathological and Psychological Distress Problems

A total of 25.9% of mothers had postpartum depression, of whom 20% were in the subclinical range and 5.9% in the clinical range. With respect to anxiety, 20.8% had state anxiety and 29.8% had trait anxiety. A total of 16% of mothers perceived severe parenting stress. Regarding social support, 3.6% of the mothers perceived low social support and 25% perceived medium social support.

### 4.3. Attachment, Reflective Functioning, and Adverse Childhood Experiences

A total of 34.9% of adolescent mothers and young mothers had a secure attachment pattern while 65.1% had an insecure attachment pattern of whom: 18.6% had an Insecure/Preoccupied attachment, 23.3% an Insecure/Dismissing attachment, 18.6% an Unresolved/Disorganized attachment and 4.7% were Not Classified.

The mean score of reflective functioning was 2.72 (SD = 1.58) which falls within the range of low reflective functioning [74].

Regarding CECA, 54.4% of adolescent mothers had at least one moderate or severe adverse childhood experience. Of these, 28.2% had one adverse experience, 14.1% had two adverse experiences, 6.4% had three adverse experiences, 5.1% had four adverse experiences and 3.8% five adverse experiences. Analyzing them in detail we find the following frequencies: 41.6% for paternal neglect, 24.4% for maternal neglect, 15.4% for maternal antipathy, 11.7% for physical abuse, 9.1% for father antipathy, 9.1% for sexual abuse and 5.1% for psychological abuse.

Considering the secondary scales of the CECA, 35.1% of the mothers lived in a conflictual family, 16.9% reported violence between parents, 19.5% had a parent with a mental disorder, 50.6% had a divorced parent, 11.7% had experienced role reversal and 7.6% had experienced the loss of a parent.

### 4.4. Mother–Infant Styles of Interaction

Analysis with the Care-Index showed that adolescent mothers had an average sensitivity score of 6.42 which was in the range at risk for relationship quality and an average score in the controlling style of 5.92 which was high according to Crittenden [77].

Infants had an average cooperative style score of 5.11 which was in the risk bracket and a high passivity score (see Table 2).

### 4.5. Maternal Mind-Mindedness

The assessment of mind-mindedness indicated at a descriptive level that the attuned mind-related comments of the adolescent mothers, M = 0.04 (SD = 0.05) were less frequent than those of the not-at-risk mothers in the Meins study [79] M = 0.10 (SD = 0.11) and the other study [80]. Adolescent and young mothers M = 0.05 (SD = 0.07) also have a higher frequency of non-attuned mind-related comments than not-at-risk mothers, M = 0.02 (SD = 0.02).

### 4.6. Correlations

We examined the associations between psychosocial and psychological risk factors and evaluated interaction styles and mind-mindedness. The psychosocial risk variable was created as the sum of low socio-economic, low education level, maternal age under 18, unwanted pregnancy, and absence of partner. With respect to the attachment style, we decided to use the AAI Coherence of the Mind scale which is considered the best indicator of the interviewee’s state of mind with respect to attachment [72,81].

Pearson’s r correlation analysis showed significant associations. Psychosocial risk was positively associated with cumulative adverse childhood experiences and negatively associated with reflective functioning and AAI Coherence of the Mind scale. Depression was significantly positively correlated with state anxiety, trait anxiety and parenting stress. State anxiety was positively correlated to trait anxiety, and parenting stress. Cumulative adverse childhood experiences were positively correlated with parental stress.

Maternal controlling style and infant compulsive–compliance style were positively correlated to maternal depression. At the level of tendency towards significance, infant difficult style was positively correlated to maternal state anxiety and negatively correlated to social support.

The AAI Coherence of mind scale was negatively correlated to cumulative adverse childhood experiences and at the level of tendency toward significance was negatively correlated to parenting stress. Reflective functioning was positively correlated to infant passive style and AAI Coherence of the Mind scale. No significant associations emerged with respect to mind-mindedness.

### 4.7. Differences Regarding Maternal Age

The sample was subdivided into mothers under 18 (*N* = 28) and mothers aged 18 to 21 (*N* = 70). The Chi-square test or *t*-test showed significant differences. Compared to mothers aged 18–21, mothers under 18 were more likely to be single without a partner (χ^2^ = 17.83; *p* = 0.000), to live in a residential mother and baby community (χ^2^ = 13.15; *p* = 0.001), to be followed by child social services (χ^2^ = 23.78; *p* = 0.000), to have been sexually abused (Fisher exact test = 8.27; *p* = 0.011) and to have a higher score on the AAI Father neglecting scale (t = 2.58; *p* = 0.011). No significant differences emerged between the two groups for the CECA scales, for the other AAI scales, mother–infant style of interaction, mind-mindedness, RF or other socio-demographic variables.

### 4.8. Multiple Regression Analysis

Multiple regression analysis was conducted to analyze the effect of risk factors on each mother and infant style of interaction and maternal mind-mindedness. The psychopathological risk variable was created as the sum of the presence of depression, state anxiety, and trait anxiety. The unresolved/disorganized AAI classification was also used to create a dichotomous variable classification of maternal disorganized/non-disorganized attachment. We tested theoretically relevant interactions and risk factors, namely psychosocial risk, psychopathological risk, AAI Coherence of Mind scale, AAI disorganization, and RF.

For the maternal sensitive style, the model explained 24% of the variance, which was not statistically significant, F (6, 31) = 1.26; *p* = 0.31. No risk factors had a significant predictive effect on maternal sensitive style. For the maternal controlling category, the model explained 32% of the variance, which was not statistically significant, F (6, 31) = 1.8; *p* = 0.14. No risk factors had a significant predictive effect on maternal sensitive style.

For the maternal unresponsive category, the model explained 26% of the variance, which was not statistically significant, F (6, 31) = 1.37; *p* = 0.26. Considering individual factors, elevated psychosocial risk had a predictive effect of a higher score on maternal unresponsive style (b = 0.79, t = 2.54; *p* = 0.017).

For the infant cooperativity category, the model explained 18% of the variance, which was not statistically significant, F (6, 31) = 1.02; *p* = 0.43. No risk factors had a significant predictive effect on infant cooperative style.

For the infant compulsive–compliance category, the model explained 23% of the variance, which was not statistically significant, F (6, 31) = 0.86; *p* = 0.53. No risk factors had a significant predictive effect. For the infant difficulty category, the model explained 34% of the variance, which was not statistically significant, F (6, 31) = 1.98; *p* = 0.11. Considering individual factors, maternal disorganization predicted higher infant difficulty behaviors (b = 0.64, t = 3.03; *p* = 0.006).

For the infant passivity category, the model explained 29% of the variance, which was not statistically significant, F (6, 31) = 1.57; *p* = 0.19. No risk factors had a significant predictive effect.

For the MM attuned mind-related comments, the model explained 60% of the variance, which was not statistically significant, F (6, 14) = 1.77; *p* = 0.23. For the MM not attuned mind-related comments, the model explained 15% of the variance, which was not statistically significant, F (6, 14) = 0.12; *p* = 0.97. No risk factors had a significant predictive effect.

## 5. Discussion

The study outlines the psychopathological and psychosocial risk profile of adolescent and young mothers and the quality of early interactions with their infants in the post-partum period. The results show that young mothers and their infants are a high-risk population with multiple social, psychological, and relationship problems. The numerous risk factors characterizing young motherhood are also interconnected, placing young mothers and their infants in a disadvantaged condition. Almost all adolescent and young mothers come from a disadvantaged socio-economic context, have a low level of education with frequent educational difficulties and consequently leave school early and have problems finding employment, therefore becoming young people who are not in employment, education or training (NEETs). A low socio-economic level was associated with a low level of education and unwanted pregnancy. The literature indicates that a low SES is a risk factor for the wellbeing of the mother, the development of the infant and their relationship [82,83].

The family context was also multiproblematic. Adolescent mothers tend to live with their parents who in turn, in almost all cases, became parents at a young age. Adolescent mothers frequently do not have a partner or do not have a stable relationship with the infant’s father. In 76% of cases the pregnancy was unwanted.

Looking at the psychopathological profile, we find that 25% of the young mothers of our sample suffer from depression, which is around twice as high as the percentage in adult mothers in non-clinical samples [84]. State and trait anxiety is also high (29%), highlighting the comorbidity between depression and anxiety in the perinatal period for young mothers just as is the case with adult mothers [85]. Correlation analysis also shows that maternal depression is associated with maternal controlling interaction style and infant compulsive-compliance style and maternal state anxiety with infant difficult style. As is well-known, maternal depression and anxiety can have a negative effect on parenting quality since depressed and anxious mothers are more emotionally detached or more intrusive in their relationship with the infant [86]. Maternal post-partum depression and anxiety can also lead to short and long-term negative consequences for the infant in the development of psychopathologies [85]. Lastly, 16% of the young mothers in our study perceive a high degree of stress in exercising the parenting role and the perception of a high degree of stress is correlated with having a number of adverse childhood experiences.

However, contrary to what was hypothesized, a high percentage of low perceived social support did not emerge. In this regard we may hypothesize that, in the first months, mothers do not yet perceive a sense of social isolation. This could, however, increase with the development and the new needs of the infant.

It is also interesting to note the psychological profiles observed: more than 60% of the adolescent and young mothers have insecure attachment and less than 40% have secure attachment, a distribution which is similar to that of the clinical samples and the at-risk samples [87], with a high percentage of unresolved/disorganized attachment. This is also connected to the result relating to adverse childhood experiences, which more than half of the sample had, in line with the studies in the literature which indicate a greater frequency of adverse experiences among adolescent mothers than among adult mothers [19,88,89]. In 30% of cases, they had suffered more than one adverse childhood experience. Examining the family history in detail it may be noted that the most frequent adverse experience is paternal neglect followed by maternal neglect. Greater neglect is found in mothers under the age of 18 than in mothers between 18–21. Adolescent mothers who have experienced abuse and neglect are also at risk for the perpetuation of such behavior (aggressive, hostile conduct and abuse) with their infants, leading to intergenerational transmission of the trauma from mother to child [26]. From a psychodynamic perspective, it may be hypothesized that adolescents who have had adverse experiences see motherhood as a potential opportunity for redemption and/or reparation of emotional deprivation and interpersonal dysfunction they had in their multiproblematic family [90].

The adolescent mothers in our sample also have low reflective functioning, which indicates their difficulty in mentalization. They have difficulty recounting and thinking in terms of mental and emotional states with respect to their experiences of attachment with their parents and consequently understanding how their, often traumatic, childhood experiences may affect their relationship with their infant. It must be observed in this regard that insecure attachment models and low maternal reflective function are associated with low sensitivity in the first years of the infant’s life and often predict infant insecure attachment which becomes a risk factor for the development of externalizing and internalizing problems [91,92].

It is also interesting to note that in our study having a greater number of psychosocial risks such as low SES, a low level of education, an absent partner and an unwanted pregnancy, correlated with a higher degree of insecure attachment, lower reflective functioning, and a higher number of adverse childhood experiences in young mothers. Psychosocial risk is therefore associated with many problematic indicators for the development of an adequate mother–infant relationship.

Concerning the quality of interaction styles in the first months, the results show that both maternal and infant behavior come within a risk profile for the relationship between the two partners. The mothers have low sensitivity and are intrusive, displaying physical and verbal aggression and negative emotions and the infants have low cooperation and are passive, with few interactive exchanges and little expression of positive emotions. Regarding capacity for mentalization, the young mothers show a low level of mind-mindedness, expressing few comments on the mental and emotional states of the infant, the majority of which are not attuned to the activity of the infant.

With respect to the age comparison of the mothers, the results indicate a few but significant differences, i.e., mothers under the age of 18 are more likely to be single, to have been sexually abused and to have been followed by child social services or to have lived in a residential mother and baby community. Mothers under the age of 18, therefore, seem to be in a more serious situation of psychosocial risk while they do not seem to present more psychopathological risks such as anxiety and depression.

Regression analysis showed that greater psychosocial risks are predictive of a maternal non-responsive style, characterized by withdrawal and emotional detachment while maternal disorganization with respect to attachment is predictive of greater infant difficult behaviors. Low maternal and infant sensitivity and maternal mind-mindedness are not predicted by a specific risk factor such as psychosocial, psychopathological, attachment model, disorganization or reflective function. However, in analyzing the variances, the regression models with the multiple risk factors as predictors, albeit not significant, had a high degree of explained variance, with a range of 18–34% for interaction styles and of 60% for attuned mind related comments. In this regard it may be hypothesized that adolescent or young motherhood is, per se, a condition of disadvantage in which there are numerous interconnected risk factors which place young mothers at risk for low sensitivity and low mentalization in initial early interaction with the infant.

This study has a number of strengths and limitations. One of the strengths is to analyze different types of risk and protective factors in a population at risk as adolescent and young mothers. Moreover, our study considers not only social and psychopathological aspects of the mother but also the quality of early interactions with the infant. The first limit is the low number of participants who completed the entire battery of tests which limits the generalizability of the results. Another limit relates to the assessment of psychopathological risk by self-report questionnaires. It would also be interesting to outline the psychosocial and psychopathological risk profile of young fathers. Future studies could compare young mothers with a sample of adult mothers in order to understand in greater detail the role of risk factors. It would also be interesting to longitudinally evaluate maternal mental health and parent-infant interaction in order to monitor the quality of the relationship and the effects on the infant’s socio-emotional development.

## 6. Conclusions and Clinical Implications

The adolescent and young mothers we studied are therefore to be considered a high-risk sample, characterized by a multiproblematic history with multiple risk factors. They also show difficulty in the first months of their infant’s life, having low sensitivity and being intrusive towards the infant and being poorly attuned to his emotional states. Psychopathological risk and the psychosocial risk factors which characterize adolescent motherhood not only can have a negative impact on short term maternal wellbeing and on mother–infant interaction but can also have a negative impact on the mother–infant relationship and on the neuropsychological and socio-emotional development of the infant in the long term. It is therefore essential to early identify all the risk factors at different social, educational, and psychological levels that characterize adolescent and young mothers, their partners and their children.

Despite the difficulties encountered by adolescent mothers, many studies, indeed, show that when they have adequate support at their disposal, they can activate their resources and improve their condition of disadvantage. Social support is therefore an important protective factor for young parents [93]. It is therefore fundamental to implement dedicated, specialized intervention in order to support young parents from pregnancy onwards and help them in taking on the new role, intervening at a number of levels: psychological, psychoeducational, social, and relational. Our findings, in fact, suggest the importance of providing integrated care to support maternal well-being and parenting with an enlarged work team, especially for mothers under 18 years old who have more risk factors and live in more critical conditions. One example of a complex and specialized intervention program is PRERAYMI (Promoting responsiveness. emotion regulation and attachment in adolescent mothers and their infants) [7,37] implemented at SAGA Service of the ASST Santi Paolo e Carlo Hospital of Milan. The intervention provides support to adolescent parents from the first stages of pregnancy up to children aged 2 years old and has been shown to be effective with regard to various critical aspects concerning adolescent motherhood, increasing maternal sensitivity and capacity for mentalization with regard to the infant, supporting the parent–infant relationship and preventing mistreatment and abuse of the infant. The implementation of interventions aimed at young mothers could also favor the reduction of social isolation, psychopathological distress, other repeated pregnancy and child abuse, thus promoting maternal well-being and positive socio-emotional development of the child.

## Figures and Tables

**Table 1:** Socio-demographic characteristics.

Adolescent and Young Mothers	
*Age* Mean (SD; range)	18.46 (1.99; 14–21)
*Marital status*	
Single	29 (30%)
Married	68 (70%)
*Living arrangements*	
with a partner	33 (35%)
with a parent	53 (57%)
residential mother and baby Community	7 (7%)
*Education*	
Elementary	38 (45%)
Middle school	29 (34%)
High school	17 (21%)
*Socio-economic level*	
Very low	22 (21.4%)
Low	64 (65.3%)
Low-Medium	13 (13.3%)
*No Job*	77 (78%)
*Young people who are not in employment, education or training (NEET)*	64 (66%)
*Unwanted pregnancy*	72 (76%)
*Residential community*	17 (17%)
*Child social services*	31 (31%)
*Parent divorce*	46 (50%)
*History of parenthood at a young age*	90 (90%)

**Table 2:** Correlations.

	(1)	(2)	(3)	(4)	(5)	(6)	(7)	(8)	(9)	M	SD
Psychosocial risk (1)	-									2.43	0.96
Depression (2)	0.09	-								6.81	4.08
State anxiety (3)	−0.06	0.56 ***	-							31.48	9.20
Trait anxiety (4)	0.06	0.60 ***	0.61 ***	-						36.29	9.04
Parenting stress (5)	0.10	0.46 *	0.42 **	0.14	-					66.68	16.99
Social support (6)	−0.10	0.01	−0.12	−0.21	−0.24	-				67.62	15.16
RF (7)	−0.25 *	−0.25	−0.28	0.00	−0.03	−0.15	-			2.73	1.58
AAI Coherence M (8)	−0.23 *	0.12	−0.15	0.13	−0.34 +	0.12	0.76 ***	-	-	4.04	1.65
Cumulative adverse childhood experiences (9)	0.27 *	0.02	−0.11	−0.09	0.67 ***	0.17	−0.13	−0.30 **	-	1.15	1.37
Sensitivity	−0.09	−0.20	−0.12	−0.12	0.08	−0.04	−0.02	−0.10	−0.11	6.42	3.14
Controlling	−0.02	0.30 *	0.21	0.24	−0.10	0.32	−0.07	0.06	−0.02	5.92	3.42
Unresponsive	0.15	−0.14	−0.12	−0.15	0.03	−0.33	0.13	0.04	0.18	1.66	2.47
Cooperative	−0.00	−0.09	−0.02	0.02	0.15	−0.00	−0.07	−0.07	−0.12	5.11	3.49
Compulsive-Compliance	0.10	0.46 **	0.02	0.13	−0.23	0.29	−0.01	−0.09	0.03	0.46	1.13
Difficulty	0.02	−0.03	0.29 +	0.24	0.06	−0.35 +	−0.14	0.12	−0.10	1.96	2.68
Passive	−0.06	0.06	−0.18	−0.25	−0.23	0.07	0.23 *	0.02	0.20	6.42	3.47
MM attuned	−0.15	−0.22	0.11	0.00	0.05	0.09	0.22	−0.05	0.09	0.04	0.05
MM non attuned	0.05	0.18	0.32	−0.02	−0.33	−0.18	−0.03	−0.14	0.21	0.05	0.11

+ *p* < 0.10, * *p* < 0.05, ** *p* < 0.01, *** *p* < 0.000.

## Data Availability

The datasets generated and/or analyzed during the current study are not publicly available but are available from the corresponding author on reasonable request.
